# Hospitalization for COVID-19, Other Respiratory Infections, and Postacute Patient-Reported Symptoms

**DOI:** 10.1001/jamanetworkopen.2024.41615

**Published:** 2024-10-25

**Authors:** Yaqing Gao, Yunhe Wang, Li Chen, Junqing Xie, Daniel Prieto-Alhambra

**Affiliations:** 1 Nuffield Department of Population Health, University of Oxford, Oxford, United Kingdom; 2 Centre for Statistics in Medicine and NIHR Biomedical Research Centre Oxford, NDORMS, University of Oxford, Oxford, United Kingdom

## Abstract

This cohort study examines patient-reported, multisystem, postacute symptoms among individuals hospitalized with COVID-19 or other lower respiratory tract infections vs those without such hospitalizations during the same period.

## Introduction

Chronic multisystem manifestations of SARS-CoV-2 infection (ie, post–COVID-19 condition [PCC]) are a specific type of postacute infection syndrome (PAIS) that can occur after other lower respiratory tract infections (LRTIs) but are often overlooked.^1^ Research on PAIS primarily captures it through inpatient diagnoses, missing milder cases and those not seeking care.^2^ We performed pairwise comparisons of patient-reported, multisystem, postacute symptoms among individuals hospitalized with COVID-19, other LRTIs, and those without LRTI hospitalizations during the same study period.

## Methods

This cohort study used data from the UK Biobank (UKB)^3^ and received ethical approval from the North West Multi-center Research Ethics Committee. All participants provided written informed consent. Additional details on the UKB are shown in the eAppendix in Supplement 1. We followed STROBE reporting guidelines.

We categorized respondents into COVID-19 hospitalization, other LRTI hospitalization, and a reference group, those without any LRTI hospitalizations (eFigure in Supplement 1). We identified COVID-19 and non–COVID-19 LRTI hospitalizations that occurred between March 1, 2020, and the survey date using *International Statistical Classification of Diseases and Related Health Problems, Tenth Revision* codes; 399 respondents hospitalized within 12 weeks of their survey date were excluded to study postacute symptoms.^4^ The index date was the latest hospitalization date for COVID-19 or other LRTI groups and the survey date for the reference group.

We used overlap weighting to balance participants’ characteristics across the 3 groups,^5^ including age at index date, sex, socioeconomic and lifestyle factors measured at UKB recruitment, and pre–index date disease history (eTable in Supplement 1). Additional details on patient exclusion and our statistical methods are shown in are shown in the eAppendix in Supplement 1.

## Results

In total, 191 710 eligible participants (mean [SD] age at index date, 69.2 [7.6] years; 109 523 female [57.1%]) were finally included: 1153 hospitalized with COVID-19, 1304 with other LRTIs, and 189 253 in the reference group. After the overlap weighting, all prespecified covariates were well balanced across the 3 groups, with standardized mean differences less than 0.1 (Table 1). Compared with the reference group, COVID-19 hospitalization was associated with higher risks of 23 of 45 symptoms (Table 2). These symptoms were observed across the ear, nose, and throat; respiratory; neurological; gastrointestinal; and musculoskeletal systems. The greatest risk was observed for ageusia (odds ratio [OR], 2.27; 95% CI, 1.87-2.75) and severe fatigue (OR, 2.18; 95% CI, 1.70-2.81). Other LRTI hospitalizations (vs reference group) were also associated with higher risks of 18 of 45 symptoms. The most prominent symptoms were related to the respiratory system (eg, pain on breathing, OR, 2.51; 95% CI, 1.99-3.17), with a modestly increased risk of gastrointestinal and neurological symptoms.

**Table 1.** Characteristics of the Study Population^a^

Characteristic	Before overlap weighting				After overlap weighting			
	Participants, No. (%)			Maximum pairwise SMD	Participants, No. (%)			Maximum pairwise SMD
	COVID-19 (n = 1153)	Other LRTIs (n = 1304)	Reference (n = 189 253)		COVID-19 (n = 1098.2)	Other LRTIs (n = 1236.4)	Reference (n = 63 456.9)	
Age, mean (SD), y^b^	69.3 (7.9)	71.0 (7.3)	69.2 (7.6)	0.24	70.3 (7.6)	70.1 (7.5)	70.1 (7.6)	0.02
Sex								
Female	508 (44.1)	631 (48.4)	108 384 (57.3)	0.27	510.3 (46.4)	568.7 (45.9)	28 043.1 (46.2)	0.01
Male	645 (55.9)	673 (51.6)	80 869 (42.7)		589.5 (53.6)	671.0 (54.1)	32 689.7 (53.8)	
Townsend Deprivation Index quintile								
1	197 (17.1)	268 (20.6)	43 621 (23.0)	0.15	205.0 (18.6)	234.3 (18.9)	11 386.5 (18.7)	0.01
2	251 (21.8)	270 (20.7)	41 128 (21.7)	0.03	239.1 (21.7)	268.0 (21.6)	12 985.7 (21.4)	0.01
3	225 (19.5)	275 (21.1)	39 227 (20.7)	0.04	222.0 (20.2)	248.6 (20.1)	12 375.9 (20.4)	0.01
4	247 (21.4)	268 (20.6)	36 738 (19.4)	0.05	225.7 (20.5)	258.2 (20.8)	12 697.5 (20.9)	0.01
5	233 (20.2)	223 (17.1)	28 539 (15.1)	0.13	208.0 (18.9)	230.6 (18.6)	11 287.2 (18.6)	0.01
Education								
Primary	139 (12.1)	148 (11.3)	13 479 (7.1)	0.17	126.4 (11.5)	147.3 (11.9)	7224.4 (11.9)	0.01
Secondary	250 (21.7)	290 (22.2)	39 994 (21.1)	0.03	239.8 (21.8)	277.0 (22.3)	13 291.3 (21.9)	0.01
Postsecondary, nontertiary	149 (12.9)	180 (13.8)	24 258 (12.8)	0.03	144.0 (13.1)	168.1 (13.6)	8066.2 (13.3)	0.01
Tertiary	615 (53.3)	686 (52.6)	111 522 (58.9)	0.13	589.6 (53.6)	647.4 (52.2)	32 150.9 (52.9)	0.03
Smoking								
Never	543 (47.1)	657 (50.4)	110 857 (58.6)	0.23	537.0 (48.8)	604.7 (48.8)	29 523.5 (48.6)	<0.01
Previous	494 (42.8)	512 (39.3)	64 922 (34.3)	0.18	446.3 (40.6)	515.3 (41.6)	25 044.4 (41.2)	0.02
Current	116 (10.1)	135 (10.4)	13 474 (7.1)	0.11	116.5 (10.6)	119.7 (9.7)	6165.0 (10.2)	0.03
Drinking^c^	264 (22.9)	310 (23.8)	42 598 (22.5)	0.03	261.0 (23.7)	290.5 (23.4)	14 231.1 (23.4)	0.01
Body mass index category^d^								
Normal (<25)	260 (22.5)	359 (27.5)	73 374 (38.8)	0.36	281.7 (25.6)	303.6 (24.5)	15 086.2 (24.8)	0.03
Overweight (25-30)	476 (41.3)	560 (42.9)	78 871 (41.7)	0.03	464.8 (42.3)	522.9 (42.2)	25 652.9 (42.2)	<0.01
Obesity (≥30)	417 (36.2)	385 (29.5)	37 008 (19.6)	0.38	353.3 (32.1)	413.3 (33.3)	19 993.7 (32.9)	0.03
Other comorbidities								
Cancer	322 (27.9)	405 (31.1)	33 401 (17.6)	0.32	321.2 (29.2)	371.3 (30.0)	17 904.5 (29.5)	0.02
Chronic obstructive pulmonary disease	131 (11.4)	193 (14.8)	5187 (2.7)	0.44	137.8 (12.5)	153.7 (12.4)	7813.2 (12.9)	0.01
Dementia	11 (1.0)	13 (1.0)	263 (0.1)	0.11	10.5 (1.0)	11.6 (0.9)	626.6 (1.0)	0.01
Stroke	75 (6.5)	67 (5.1)	3709 (2.0)	0.23	62.4 (5.7)	73.0 (5.9)	3515.4 (5.8)	0.01
Asthma	259 (22.5)	353 (27.1)	26 366 (13.9)	0.33	264.1 (24.0)	302.1 (24.4)	14 859.3 (24.5)	0.01
Chronic kidney disease	137 (11.9)	168 (12.9)	6500 (3.4)	0.35	131.9 (12.0)	157.4 (12.7)	7470.9 (12.3)	0.02
Depression	212 (18.4)	224 (17.2)	19 839 (10.5)	0.23	198.3 (18.0)	220.1 (17.8)	10 810.2 (17.8)	0.01
Diabetes	195 (16.9)	205 (15.7)	10 080 (5.3)	0.37	173.3 (15.8)	207.7 (16.8)	9868.2 (16.2)	0.03
Hypertension	618 (53.6)	747 (57.3)	61 353 (32.4)	0.52	602.9 (54.8)	690.5 (55.7)	33 516.0 (55.2)	0.02
Ischemic heart disease	199 (17.3)	293 (22.5)	13 328 (7.0)	0.45	213.9 (19.4)	238.9 (19.3)	11 872.8 (19.5)	0.01
Rheumatoid arthritis	87 (7.5)	81 (6.2)	3489 (1.8)	0.27	72.6 (6.6)	82.9 (6.7)	4186.4 (6.9)	0.01

Abbreviations: LRTI, lower respiratory tract infection; SMD, standardized mean difference.

^a^ Baseline characteristics were stratified by 3 comparison groups: COVID-19 hospitalization, other contemporary LRTI hospitalization, and reference group (those without LRTI hospitalizations).

^b^ Age at index date refers to the latest hospitalization date for COVID-19 and/or other LRTI groups and the survey date for the reference group.

^c^ Refers to drinking daily or almost daily.

^d^ Body mass index is calculated as weight in kilograms divided by height in meters squared.

**Table 2.** Association of COVID-19 Hospitalization, Other LRTI Hospitalization, and Reference Group (No LRTI Hospitalization) With 45 Patient-Reported Physical and Psychological Symptoms^a^

Category and symptoms	OR (95% CI)		
	COVID-19 vs reference	Other LRTIs vs reference	COVID-19 vs other LRTIs
Ear, nose, and throat			
Loss or change in sense of smell	2.04 (1.69-2.47)^b^	1.08 (0.86-1.36)	1.80 (1.34-2.42)^b^
Loss or change in sense of taste	2.27 (1.87-2.75)^b^	1.42 (1.14-1.77)	1.52 (1.13-2.03)^b^
Hearing loss	1.16 (1.02-1.33)	1.22 (1.08-1.38)^b^	0.94 (0.79-1.13)
Tinnitus	0.99 (0.86-1.14)	1.05 (0.92-1.20)	0.95 (0.78-1.15)
Other hearing issues	0.92 (0.68-1.24)	1.46 (1.16-1.83)	0.61 (0.42-0.89)^b^
Vision problems	1.12 (0.96-1.30)	1.27 (1.11-1.46)^b^	0.87 (0.71-1.07)
Nasal congestion	0.89 (0.76-1.04)	0.99 (0.85-1.15)	0.87 (0.70-1.09)
Sore or painful throat	1.07 (0.83-1.39)	1.19 (0.94-1.50)	0.89 (0.63-1.27)
Respiratory and chest			
Shortness of breath or trouble breathing	1.93 (1.69-2.22)^b^	2.27 (2.01-2.57)^b^	0.86 (0.71-1.03)
Postural tachycardia	2.16 (1.71-2.73)^b^	1.32 (1.01-1.74)	1.60 (1.12-2.29)^b^
Tightness in the chest	1.55 (1.25-1.91)^b^	2.02 (1.68-2.41)^b^	0.76 (0.58-1.01)
Chest pressure	1.39 (1.08-1.79)	1.66 (1.33-2.07)^b^	0.87 (0.62-1.21)
Chest pain	1.58 (1.23-2.02)^b^	1.94 (1.56-2.40)^b^	0.79 (0.57-1.09)
Heart issues	1.43 (1.22-1.68)^b^	1.53 (1.32-1.77)^b^	0.93 (0.75-1.15)
Pain on breathing	2.05 (1.56-2.69)^b^	2.51 (1.99-3.17)^b^	0.79 (0.55-1.12)
Persistent cough	1.24 (1.04-1.48)	1.74 (1.50-2.02)^b^	0.72 (0.57-0.90)^b^
Phlegm production or a chest cough	1.04 (0.88-1.22)	1.74 (1.53-1.99)^b^	0.60 (0.49-0.74)^b^
Neurological			
Problems thinking	1.65 (1.46-1.87)^b^	1.21 (1.07-1.37)	1.36 (1.14-1.62)^b^
Problems communicating	1.33 (1.12-1.57)^b^	1.23 (1.05-1.45)	1.07 (0.85-1.35)
Numbness or tingling somewhere in the body	1.40 (1.23-1.61)^b^	1.20 (1.05-1.37)	1.17 (0.97-1.41)
Dizziness or light headedness	1.39 (1.20-1.62)^b^	1.30 (1.13-1.51)^b^	1.05 (0.86-1.30)
Headaches	1.30 (1.09-1.56)	1.20 (1.01-1.43)	1.11 (0.86-1.42)
Problems relating to mood anxiety and emotions	1.24 (1.09-1.41)	1.15 (1.02-1.31)	1.07 (0.89-1.29)
Gastrointestinal			
Gastrointestinal issues	1.41 (1.22-1.63)^b^	1.37 (1.20-1.57)^b^	1.01 (0.83-1.23)
Abdominal pain or stomachache	1.37 (1.14-1.66)^b^	1.48 (1.24-1.76)^b^	0.91 (0.70-1.17)
Nausea and/or vomiting	1.58 (1.21-2.07)^b^	1.71 (1.34-2.19)^b^	0.90 (0.63-1.29)
Decrease in appetite	1.40 (1.18-1.66)^b^	1.64 (1.41-1.91)^b^	0.83 (0.66-1.04)
Musculoskeletal			
Joint pain or swelling of joints	1.10 (0.97-1.24)	0.99 (0.88-1.11)	1.11 (0.94-1.32)
Leg pain	1.27 (1.12-1.45)^b^	1.20 (1.06-1.36)	1.05 (0.88-1.26)
Neck pain or stiff neck	1.14 (0.99-1.31)	1.14 (1.00-1.30)	0.99 (0.82-1.20)
Muscle pain or achy muscles	1.28 (1.13-1.45)^b^	1.18 (1.05-1.33)	1.07 (0.90-1.27)
Bone pain	1.52 (1.30-1.78)^b^	1.15 (0.98-1.35)	1.33 (1.06-1.67)^b^
Back pain	1.09 (0.96-1.24)	1.10 (0.98-1.24)	0.98 (0.82-1.17)
Dermatological and allergic			
Red or purple sores or blisters on feet	1.34 (0.92-1.96)	1.24 (0.85-1.79)	1.05 (0.62-1.78)
Skin issues raised red itchy areas new rash	1.11 (0.95-1.30)	1.11 (0.96-1.29)	1.01 (0.81-1.26)
New allergy or intolerance	0.90 (0.59-1.38)	0.96 (0.65-1.42)	0.90 (0.50-1.61)
General systemic			
Weakness of muscles or difficulty moving arms and legs	1.71 (1.49-1.97)^b^	1.51 (1.32-1.73)^b^	1.11 (0.92-1.35)
Mild fatigue	1.61 (1.43-1.82)^b^	1.34 (1.20-1.51)^b^	1.19 (1.01-1.40)^b^
Severe fatigue	2.18 (1.70-2.81)^b^	1.40 (1.05-1.86)	1.49 (1.02-2.17)^b^
Unrestful sleep	1.07 (0.94-1.21)	1.04 (0.93-1.17)	1.03 (0.87-1.22)
Difficulty sleeping	1.08 (0.95-1.23)	1.01 (0.89-1.14)	1.06 (0.89-1.27)
Postexertional symptom exacerbation	1.84 (1.59-2.13)^b^	1.68 (1.46-1.94)^b^	1.09 (0.89-1.33)
Night sweats	1.19 (1.03-1.39)	1.09 (0.94-1.26)	1.09 (0.88-1.35)
Fever	1.13 (0.78-1.62)	1.43 (1.05-1.94)	0.79 (0.50-1.27)
Chills or feeling too cold	1.39 (1.02-1.89)	1.47 (1.11-1.95)	0.91 (0.60-1.37)

Abbreviations: LRIT, lower respiratory tract infection; OR, odds ratio.

^a^ Pairwise comparisons were made of 45 patient-reported symptoms among individuals hospitalized with COVID-19, other contemporary LRTIs, and reference group (those without LRTI hospitalizations). Logistic regression was used to estimate the ORs of symptoms for each pair of comparison. Estimates for the comparison between infection groups (COVID-19 and other LRTI hospitalization) and the reference group (those without any LRTI hospitalization) are corrected for multiple testing based on Bonferroni method.

^b^ *P* < .05.

Compared with other LRTIs, COVID-19 hospitalizations were associated with an increased risk of 7 individual symptoms: anosmia (OR, 1.80; 95% CI, 1.34-2.42), ageusia (OR, 1.52; 95% CI, 1.13-2.03), postural tachycardia (OR, 1.60; 95% CI, 1.12-2.29), problem of thinking (OR, 1.36; 95% CI, 1.14-1.62), bone pain (OR, 1.33; 95% CI, 1.06-1.67), mild fatigue (OR, 1.19; 95% CI, 1.01-1.40), and severe fatigue (OR, 1.49; 95% CI, 1.02-2.17). Conversely, persistent chest cough was less common for COVID-19.

## Discussion

This cohort study found that PAIS is not unique to COVID-19; it can also occur in people with other severe LRTIs. However, compared with other LRTIs, COVID-19 appeared to impose an extra burden of neurological, cognitive, and fatigue symptoms. These findings highlight the similarities and differences between PCC and PAIS triggered by other pathogens, which will inform tailored clinical management and offer mechanistic insights into these previously overlooked syndromes. Limitations include potential residual confounding, the healthier profile of UKB participants vs the general population, and the lack of repeated-measure data for symptom trajectories.

## Notes

Supplement 1. **eAppendix.** Supplemental methods **eFigure.** Study design **eTable.** Definition of covariates
eReferences

Supplement 2.
Data Sharing Statement
