# Supplementary material for: Hospitalization for COVID-19, Other Respiratory Infections, and Postacute Patient-Reported Symptoms
Source: JAMA Netw Open. 2024 Oct 25;7(10):e2441615. doi: 10.1001/jamanetworkopen.2024.41615 (PMC11581579; doi:10.1001/jamanetworkopen.2024.41615)
Supplement: Supplement 1. — eAppendix. Supplemental methods eFigure. Study design eTable. Definition of covariates eReferences [file jamanetwopen-e2441615-s001.pdf]

Supplemental Online Content

Gao Y, Wang Y, Chen L, Xie J, Prieto-Alhambra D. Hospitalization for COVID-19, other respiratory infections, and postacute patient-reported symptoms. *JAMA Netw Open*. 2024;7(10):e2441615.
doi:10.1001/jamanetworkopen.2024.41615

eAppendix. Supplemental methods
eFigure. Study design
eTable. Definition of covariates
eReferences

This supplemental material has been provided by the authors to give readers additional information about their work.

© 2024 Gao Y et al. *JAMA Network Open.*

eAppendix. Supplemental methods

The UK Biobank (UKB) is a prospective cohort study that recruited over 500 000 people aged 40 to 69 years across the UK between 2006 and 2010. During the COVID-19 pandemic, UKB invited approximately 333 000 participants to complete the Health and Well-being Survey, assessing their current experience of 45 symptoms selected by PCC experts that are rarely recorded in routine health care data. A total of 195 680 individuals (approximately 60% response rate) completed the survey between June 2022 and May 2023.

A total of 3582 respondents with missing covariate data were excluded. The risks of 45 symptoms were compared using weighted logistic regression, and we additionally adjusted for the time gaps between index and survey dates in comparisons between COVID-19 and other LRTI groups.

Statistical significance was defined as 2-tailed $P < .05$. Data were analyzed using R statistical software version 4.2.2 (R Project for Statistical Computing).

© 2024 Gao Y et al. *JAMA Network Open.*

eFigure. Study design

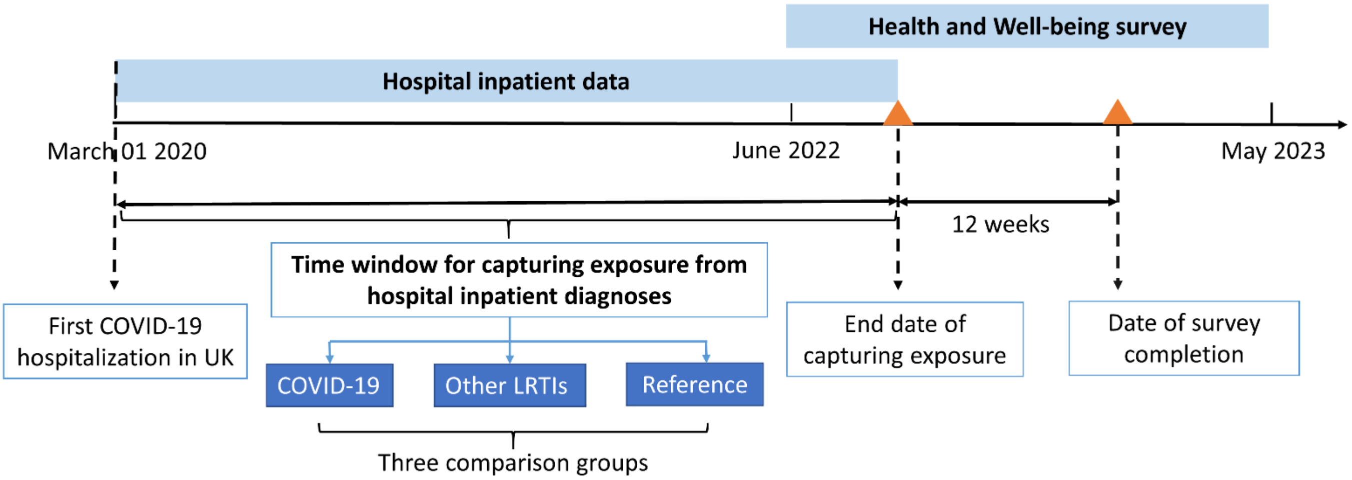

LRTIs: Lower respiratory tract infections

For each participant in the Health and Well-being survey, assuming that the orange triangle mark represented their survey completion date, the time window for capturing exposure from hospital inpatient records (hospitalizations for COVID-19, other lower respiratory tract infections (LRTIs), and no LRTI-related hospitalization [reference group]) extended from March 1, 2020, to 12 weeks before the survey completion date. Since patients could have multiple hospital-treated infections during that time period, participants hospitalized for both COVID-19 and other LRTIs were categorized based on their most recent diagnosis. If both diagnoses occurred on the same date, they were classified under COVID-19. The ICD-10 codes for COVID-19 (B34.2, B97.2, U07.1, U07.2, and U10) and other LRTIs (J09-J22) aligned with previous studies.[1,2] Individuals hospitalized with COVID-19 or other LRTIs within 12 weeks before the survey, or those with missing covariate data, were excluded.

© 2024 Gao Y et al. *JAMA Network Open.*

eTable. Definition of covariates

Variable	UK Biobank field ID(s)	Measurement item(s)	Definition/categorization
Age	34	(Registry) year of birth	Age at index date = year of index date – year of birth
Sex	31	Registry	
Ethnicity	21000	(Questionnaire) amalgam of sequential branching questions	We used the first non-missing value across all measurement occasions.<br>1) White;<br>2) Non-white (Asian or Asian British, Black or Black British, Chinese, Mixed, and other ethnic group)
Education	6138	(Questionnaire) self-reported qualifications	Participants with multiple qualifications were assigned the highest qualification attained, which was subsequently converted to the International Standard Classification for Education (ISCED) code representing years of education: [3,4]<br>1) Tertiary: College/University Degree, National Vocational Qualification (NVQ) / Higher National Diploma (HND) / Higher National Certificate (HNC) or equivalent<br>2) Post-secondary non-tertiary: Other professional qualifications (e.g., nursing, teaching)<br>3) Secondary: General Certificate of Education Advanced Level (A levels) / General Certificate of Education Advanced Subsidiary Level (AS levels) or equivalent, General Certificate of Education Ordinary Level (O levels) / General Certificate of Secondary Education (GCSEs) or equivalent, Certificate of Secondary Education (CSEs) or equivalent<br>4) Primary: None of the above
Townsend deprivation index (TDI)	189	The TDI was computed for each participant using the national census data preceding their enrolment in UK Biobank. The score was assigned based on the output area of the participant's postcode.	A greater Townsend index score implies a greater degree of deprivation. The entire UK Biobank sample was categorized into quintiles based on their TDI, with quintile 1 representing the richest and quintile 5 representing the poorest.
Smoking status	20116	(Questionnaire) self-reported smoking-status	1) Never;<br>2) Previous;<br>3) Current
Alcohol consumption	1558	(Questionnaire) self-reported drinking frequency	1) ≤ 4 times week;<br>2) Daily or almost daily

© 2024 Gao Y et al. *JAMA Network Open.*

<table>
<tr><td>Body mass index (BMI)</td><td>21001</td><td>(Physical measures) height and weight</td><td>The classification of BMI is utilized by the World Health Organization:[5]
1) Normal (<25 kg/m^2);
2) Overweight (25–30 kg/m^2);
3) Obese (≥30 kg/m^2)</td></tr>
<tr><td>Medical history</td><td>Category 100092, 20001, Category 1712</td><td>First occurrences, Cancer register
(Verbal Interview) self-reported cancer</td><td>We adjusted for disease history before most recent hospitalization (for the infection group) / survey completion (for the reference group). The UK Biobank “First occurrences” data integrated diagnosis records from various sources, including self-reported data from verbal interviews, primary care records, hospital inpatient records, and death registries. These records were standardized into 3-digit ICD-10 codes, and information was extracted regarding the date of the first recording of the code from any of the mentioned sources.
ICD codes for diseases included in this study:
<table>
<tr><td>Chronic obstructive pulmonary disease</td><td>J43, J44</td></tr>
<tr><td>Cancer</td><td>C00-C97, O01, 140-208 (ICD-9), 630 (ICD-9)</td></tr>
<tr><td>Chronic kidney disease</td><td>N18</td></tr>
<tr><td>Ischaemic heart disease</td><td>I21, I22, I23, I24, I25</td></tr>
<tr><td>Depression</td><td>F32, F33</td></tr>
<tr><td>Diabetes</td><td>E10, E11, E13</td></tr>
<tr><td>Hypertension</td><td>I10</td></tr>
<tr><td>Stroke</td><td>I60, I61, I63, I64</td></tr>
<tr><td>Asthma</td><td>J45, J46</td></tr>
<tr><td>Dementia</td><td>F00, F01, F02, F03, G30</td></tr>
<tr><td>Rheumatoid arthritis</td><td>M05, M06</td></tr>
</table></td></tr>
</table>

© 2024 Gao Y et al. *JAMA Network Open.*

eReferences

1. Daugherty SE, Guo Y, Heath K, et al. Risk of clinical sequelae after the acute phase of SARS-CoV-2 infection: retrospective cohort study. *BMJ* 2021; **373**: n1098.
2. Taquet M, Sillett R, Zhu L, et al. Neurological and psychiatric risk trajectories after SARS-CoV-2 infection: an analysis of 2-year retrospective cohort studies including 1 284 437 patients. *Lancet Psychiatry* 2022; **9**(10): 815-27.
3. Lee JJ, Wedow R, Okbay A, et al. Gene discovery and polygenic prediction from a genome-wide association study of educational attainment in 1.1 million individuals. *Nature Genetics* 2018; **50**(8): 1112-21.
4. Carter AR, Gill D, Davies NM, et al. Understanding the consequences of education inequality on cardiovascular disease: mendelian randomisation study. *Bmj* 2019; **365**: l1855.
5. World Health Organization. A healthy lifestyle - WHO recommendations. May 2010. https://www.who.int/europe/news-room/fact-sheets/item/a-healthy-lifestyle---who-recommendations (accessed February 6 2023).

© 2024 Gao Y et al. *JAMA Network Open.*
