# Supplementary material for: Hospitalization for COVID-19, Other Respiratory Infections, and Postacute Patient-Reported Symptoms
Source: JAMA Netw Open. 2024 Oct 25;7(10):e2441615. doi: 10.1001/jamanetworkopen.2024.41615 (PMC11581579; doi:10.1001/jamanetworkopen.2024.41615)
Supplement: Supplement 2. — Data Sharing Statement [file jamanetwopen-e2441615-s002.pdf]

Data Sharing Statement

Gao. Hospitalization for COVID-19, Other Respiratory Infections, and Postacute Patient-Reported Symptoms. *JAMA Netw Open*. Published October 28, 2024.
doi:10.1001/jamanetworkopen.2024.41615

Data
Data available: Yes
Data types: Deidentified participant data
How to access data: https://www.ukbiobank.ac.uk/
When available: beginning date: 10-01-2023

Supporting Documents
Document types: None

Additional Information
Who can access the data: Researchers whose proposed use of the data has been approved
Types of analyses: For a specified purpose
Mechanisms of data availability: After approval of a proposal
